# Preparation and Characterization of Site-Specific Fatty Chain-Modified Recombinant Human Granulocyte Colony Stimulating Factor

**DOI:** 10.3389/fbioe.2022.923059

**Published:** 2022-05-23

**Authors:** Xu-Dong Wang, Wei-Jia Yu, Jia-Hui Liu, Jie Du, Kang-Nan Chen, Qin-Qin Hu, Wen-Long Sun, Guo-Qing Ying

**Affiliations:** ^1^ College of Pharmaceutical Science, Zhejiang University of Technology, Hangzhou, China; ^2^ Institute of Biomedical Research, School of Life Sciences, Shandong University of Technology, Zibo, China

**Keywords:** rhG-CSF, recombinant human granulocyte colony stimulating factor, fatty chain, fatty chain modification, site-specific modification, thiol modification, long-acting rhG-CSF

## Abstract

The clinical use of recombinant human granulocyte colony-stimulating factor (rhG-CSF) is limited by its short serum half-life. In this study, a long-acting strategy for site-specific modification of rhG-CSF with 1-pentadecyl-1H-pyrrole-2,5-dione (C15 fatty chain-maleimide, C15-MAL) was studied in mixed DMSO-aqueous solutions. The factors influencing the conjugation reaction were investigated and optimized, and a high yield of the desired product (C15-rhG-CSF) was achieved. Subsequently, C15-rhG-CSF product was efficiently purified using preparative liquid chromatography, and further characterized. Circular dichroism spectroscopy analysis showed that the secondary structure of C15-rhG-CSF had no significant difference from unmodified rhG-CSF. C15-rhG-CSF retained 87.2% of *in vitro* bioactivity of unmodified rhG-CSF. The pharmacokinetic study showed that the serum half-life of C15-rhG-CSF in mice was 2.08-fold longer than that of unmodified rhG-CSF. Furthermore, C15-rhG-CSF by single-dose subcutaneous administration showed better *in vivo* efficacy than those of both PEG_10k_-rhG-CSF by single-dose administration and rhG-CSF by multiple doses administration. This study demonstrated the potential of C15-rhG-CSF being developed into a novel drug candidate as well as an efficient process for the development of long-acting protein and peptide drugs.

## 1 Introduction

Recombinant human granulocyte colony-stimulating factor (rhG-CSF) is mainly produced from *Escherichia coli*, which has 175 amino acid residues with a molecular weight of 18.8 kDa (Liongue et al., 2009). Recently, rhG-CSF is mainly used in clinical adjuvant therapy for patients receiving chemotherapy or radiotherapy (Crea et al., 2009). However, it has a short *in vivo* half-life, resulting in frequent injection and low patient compliance (Akizawa et al., 1995). To overcome these problems, the long-acting technologies such as polyethylene glycol modification (PEGylation) (Wu et al., 2015), protein fusion (Xu et al., 2015; Do et al., 2017), and new dosage forms technologies (Kim and Park, 2004) have been used for the development of long-acting rhG-CSF. Among these technologies, PEGylation is one of the most successful methods, and several PEGylation rhG-CSF products (e.g., Pegfilgrastim (Piedmonte and Treuheit, 2008)) have been approved for clinical use. However, PEGylation proteins have some limits, such as a significant activity loss of parent proteins (Fontana et al., 2008), immunogenicity inducing by anti-PEG antibodies (Zhang et al., 2016; Shimizu et al., 2018) and metabolic safety problem due to PEG accumulating in the liver (Verhoef and Anchordoquy, 2013). PEGylated rhG-CSF, as one of PEGylation proteins, may suffer from similar issues with its increasing use. Therefore, novel strategies to develop more effective and safer long-acting rhG-CSF derivatives remain desirable.

Alternatively, fatty chain modification technology, coupling a small molecular albumin binding moiety of fatty chain to proteins or peptides (Menacho-Melgar et al., 2019), has recently received much attention due to its unique benefits as follows: 1) The coupled fatty chain in proteins and peptides can reversibly bind to human serum albumin (HSA) (Plum et al., 2013), which has an inherently long serum half-life (more than 2 weeks) (Sleep et al., 2013) because of reduced renal filtration and evasion from intracellular degradation *via* Fc receptor (FcRn)-mediated recycling (Cho et al., 2020); 2) Fatty chain is a small molecule, which cannot significantly reduce the activity of parent proteins due to its minimal steric hindrance effect (Lim et al., 2013). 3) Fatty chain is a biocompatible endogenous molecule, which has minimal immunogenicity (Schultz et al., 2018); 4) Fatty chain is a lipophilic biomolecule, which can enhanced cellular and intestinal mucosal penetration (Lau et al., 2015). Therefore, fatty chain modification technology has recently achieved great success in the development of long-acting protein and peptide drugs, and several related products (e.g., Detemir (Beji et al., 2020), Degludec (Atkin et al., 2015), Liraglutide (Tamborlane et al., 2019) and Semaglutide (Christou et al., 2019)) have been marketed. To date, the development of long-acting rhG-CSF using fatty chain modification technology has not been reported, which is worth exploring. On the other hand, the mainstream strategy for fatty chain modification at the lysine residues of proteins and peptides is not suitable for rhG-CSF, because rhG-CSF has four lysine residues (Lys17, Lys24, Lys35 and Lys41) (Behi et al., 2018). This modification strategy will result in heterogeneous conjugates, a low yield of the desired product and a significant challenge of downstream processing (Chae et al., 2010a; Chae et al., 2010b). Thus, the site-specific modification strategy is more preferred. One potential strategy is site-specific modification of the thiol group of a single cysteine residue since rhG-CSF has a unique free cysteine residue (Cys18) (Behi et al., 2018).

**FIGURE 1:**
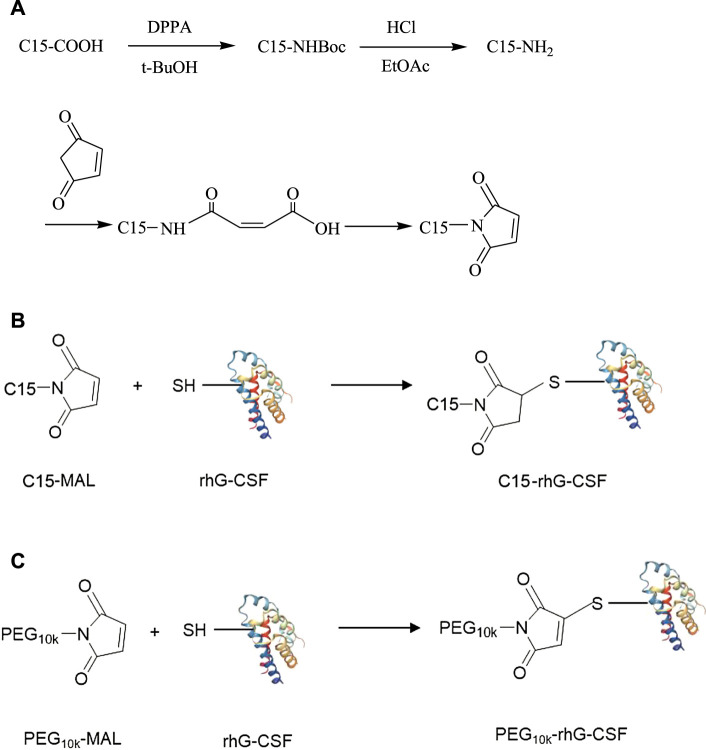
The synthetic routes of C15-MAL **(A)**, C15-rhG-CSF **(B)** and PEG_10k_-rhG-CSF **(C)**.

Taken together, the aim of this study was to develop a novel fatty chain modification strategy for the site-specific conjugation of the thiol group of Cys18 in rhG-CSF. Firstly, 1-Pentadecyl-1H-pyrrole-2,5-dione (C15 fatty chain-maleimide, C15-MAL) (Figure 1A) was synthesized from palmitic acid, which has a high affinity with HSA and has been used to prolong serum half-life of proteins and peptides in many previous studies (Hashizume et al., 1992; Lim et al., 2013; Cho et al., 2017). Secondly, modification of rhG-CSF with C15-MAL (Figure 1B) was performed in mixed DMSO-phosphate buffer solution and the reaction condition was optimized. Finally, the desired product was purified and characterized. Besides, PEGylatied rhG-CSF (Figure 1C) was also prepared as a control to compare the characteristics with fatty chain-modified rhG-CSF.

## 2 Materials and Methods

### 2.1 Materials

Recombinant human granulocyte colony stimulating factor (rhG-CSF) and the murine myeloblastic NFS-60 cell lines were obtained from Jiuyuan Gene Engineering Co., Ltd. (Hangzhou, China). Palmitic acid was purchased from Sangon Biotech (Shanghai, China). Monomethoxyl polyethylene glycols maleimide with Mw of 10 kDa (mPEG_10k_-MAL) was purchased from Aladdin (Shanghai, China). Acetonitrile of HPLC grade and trifluoroacetic acid (TFA) was purchased from Sigma-Aldrich Corporation (St. Louis, MO). All other chemicals used were analytical grade.

### 2.2 Animals

KM mice (male, 16–20 g) were purchased from the Experimental Animal Center of Shandong (Jinan, China). The license number is scxk 2014–0007. The animal husbandry system was maintained under standard laboratory conditions. All animal experimental protocols were approved by the Animal Ethics Committee of Shandong University of Technology and operated according to the guideline for the care and use of laboratory animals of Shandong University of Technology.

### 2.3 Synthesis of 1-Pentadecyl -1H-Pyrrole-2,5-Dione (C15 Fatty Chain-Maleimide, C15-MAL)

The synthetic route of C15-MAL was shown in Figure 1A. In the first step, palmitic acid (C15-COOH, 34.17 mmol) was dissolved in 60 ml tetrahydrofuran solution, and then triethylamine (34.17 mmol) and diphenyl azidophosphate (DPPA, 34.17 mmol) were added to the solution. The reaction was carried out at room temperature for 48 h under a nitrogen environment. Subsequently, the reaction mixture was treated by vacuum distillation to remove the solvent at 40°C, and then 60 ml tert-butanol (t-BuOH) was added and heated reflux at 90°C for 5 h under a nitrogen environment. After the reaction, the solvent was removed by vacuum distillation at 60°C, and then 4 mol/L HCl was added to the reaction mixture and stirred at room temperature overnight. Subsequently, ethyl acetate (EtOAc) of equal volume was added to isolate the 1-aminopentadecane (C15-NH_2_), and finally the solid powder of pure C15-NH_2_ was obtained by vacuum distillation to remove the solvent, and drying. In the second step, C15-NH_2_ (23.45 mmol) and maleic anhydride (23.45 mmol) were dissolved in 15 ml anhydrous dichloromethane. The solution was heated reflux at 40°C for 30 min. Subsequently, the reaction mixture was cooled to room temperature, and the solid was filtered off, washed with cooled dichloromethane and dried to obtain the solid powder of n-pentadecyl maleic acid. Then, n-pentadecyl maleic acid (12.8 mmol) and sodium acetate (8.8 mmol) were dissolved in 15 ml acetic anhydride and heated reflux at 100°C for 2 h. After the reaction mixture was cooled to room temperature and added with 50 ml water, the solution was transferred to a separating funnel, and then successively washed with ethyl ether (twice, each 30 ml), 2% KOH solution (twice, each 30 ml) and water (once, 30 ml). The organic phase containing C15-MAL was vacuum distilled to remove the solvent, and then the residue solid was re-dissolved in 20 ml dichloromethane, dried over MgSO_4_. Finally, the solid of crude C15-MAL was obtained by vacuum distillation to remove the solvent.

The crude C15-MAL was further purified using a YMC-Pack ODS-A C18 semi-preparative column (20 × 250 mm, 10 μm, YMC, Kyoto, Japan) on a Shimadzu SPD-M20 A system (Shimadzu, Japan). The mobile phase A and B was pure water and acetonitrile, respectively. The column was equilibrated with 90% (v/v) B, followed by isocratic elution with 90% B over 30 min at a flow rate of 15 ml/min. The effluent was detected at 215 nm and the loading volume was 2 ml for each injection.

The purity of the purified C15-MAL was evaluated by RP-HPLC analysis. The sample was injected into a Phenomenex C18 (250 mm × 4.6 mm, 5 µm) and purified using an isometric elution 90% phase B (acetonitrile and 0.1% TFA) over 60 min, phase A (water, 0.1% TFA) at a flow rate of 1.0 ml/min with UV detection at 215 nm.

The molecular weight of C15-MAL was determined by mass spectrometry using Thermo ScientificITQ 1100TM (ThermoFisher, United States). The chemical shifts of hydrogen atoms in C15-MAL were detected by ^1^H NMR, the sample to be tested was dissolved in methanol. The experimental data were recorded by 500 MHz nuclear magnetic resonance (NMR).

### 2.4 Conjugation of rhG-CSF With C15-MAL

rhG-CSF (1.0 mg/ml) dissolved in 20 mM sodium phosphate buffer (pH 6.5, 7.0, 7.5 or 8.0) and C15-MAL of the desired concentration (the molar ratio of C15-MAL to rhG-CSF of 8:1, 5:1, 3:1 or 1:1) dissolved in DMSO solution were mixed at a volume ratio of DMSO/phosphate buffer of 12:1, 10:1, 8:1, 6:1 or 4:1, and then, the reaction was carried out at temperature of 4, 20, 30 or 40°C for 0–120 min with a stirring speed of 200 rad/min. The standard condition for the analysis of each factor was as follows: volume ratio of DMSO/phosphate buffer of 10:1, mole ratio of C15-MAL to rhG-CSF of 5:1, pH 7.0, temperature 20°C and reaction time 30 min. Each sample (1 ml), taken at different time interval, was added with 50 μL of 1 M DTT to stop the reaction, and then, immediately analyzed by RP-HPLC to calculate the concentration of the desired product (C15-rhG-CSF) based on the peak area. To evaluate the conjugation efficiency, the yield of C15-rhG-CSF was calculated by Eq. 1.

$$\mathrm{Yield}\;\mathrm{of}\;C15-\mathrm{rhG}-\mathrm{CSF}\left(\%\right)=\frac{C15-\mathrm{rhG}-\mathrm{CSF}\left(\mathrm{mol}\right)}{\mathrm{The}\;\mathrm{initial}\;\mathrm{rhG}-\mathrm{CSF}\left(\mathrm{mol}\right)}\times 100$$ (1)

### 2.5 Purification of C15-rhG-CSF

The crude C15-rhG-CSF was purified using a Sepax Bio-C8 RP-HPLC column (21.2 × 250 mm, 10 μm, 300 Å, Sepax Technologies Inc., United States) on a Shimadzu SPD-M20 A system (Shimadzu, Japan). The mobile phase A was pure water with TFA adjusting pH 4.2, and mobile phase B was pure acetonitrile with TFA adjusting pH 4.2. The column was equilibrated with 40% (v/v) B, followed by elution with a linear gradient of 40%–55% B over 5 min and 55%–85% B over 30 min. The column temperature was maintained at 30°C and the flow rate was 10 ml/min, and the effluent was monitored at 280 nm. The collected fraction of the purified C15-rhG-CSF was diluted and dialyzed with 10 mM acetate buffers (pH 4.2), and then concentrated using Amicon Ultra-15 centrifugal filter devices with a 3 kDa MW cut-off (Millipore Corp. Bedford, MA, United States), and stored at −20°C until use.

### 2.6 Preparation of PEGylated rhG-CSF

PEGylated rhG-CSF was prepared as control, referring to a previously reported method (Veronese et al., 2007). Briefly, rhG-CSF was dissolved in 0.05 M sodium phosphate buffer, pH 7.2, containing 4 M guanidinium chloride (Gu HCl). And, 10 kDa PEG-MAL was added at a mole ratio of PEG-MAL to rhG-CSF of 5:1. The solution was incubated at 30°C for 2 h. Before purification, the reaction mixture was dialyzed with buffer A (10 mM acetate buffers, pH 4.2), and then concentrated using Amicon Ultra-15 centrifugal filter devices with a 3 kDa MW cut-off (Millipore Corp. Bedford, MA, United States). Subsequently, the treated reaction mixture of 2 ml was loaded on a 5 ml HiTrap CM FF column (GE Healthcare, United States) pre-equilibrated with buffer A, using an AKTA purifier 10 system (GE Healthcare, United States) at room temperature. After the column was washed with three column volumes of the buffer A, PEGylated rhG-CSF fractions were eluted with a linear salt gradient from 0 to 50% buffer B (10 mM acetate buffer containing 1 M NaCl, pH 4.2) over 120 min, at a flow rate of 1 ml/min with UV detection at 280 nm. The collected fraction of purified PEG_10k_-rhG-CSF was dialyzed with buffer A, and then concentrated using Amicon Ultra-15 centrifugal filter devices with a 3 kDa MW cut-off (Millipore Corp. Bedford, MA, United States), and stored at −20°C until use.

### 2.7 Matrix-Assisted Laser Desorption/Ionization Time-of-Flight Mass Spectroscopy Analysis

The molecular weights of rhG-CSF and the purified C15-rhG-CSF were detected by MALDI-TOF-MS on a Brukerul Traflex III TOF-MS spectrometer (Bruker Daltonics, United States) with a nitrogen laser. Each sample was spotted onto a 100-well sample plate and the R-cyano-4-hydroxycinnamic acid was the matrix. All spectra were acquired in a linear mode, and positive ions were monitored.

### 2.8 Circular Dichroism Spectroscopy Analysis

The secondary structures of rhG-CSF, PEG_10k_-rhG-CSF and C15-rhG-CSF were determined on the J-815 circular dichroism spectropolarimeter (JASCO, Japan). Each sample was adjusted to a rhG-CSF equivalent concentration of 0.2 mg/ml with 10 mM acetate buffers, pH 4.2. Spectra were recorded at wavelengths between 190 and 250 nm using a 0.1 mm path length cell at 25°C. Each spectrum was obtained from an average of three scans, and CD spectra were corrected for buffer contributions.

### 2.9 RP-HPLC Analysis

The reaction mixtures derived from the modification of rhG-CSF with C15-MAL and the purified C15-rhG-CSF were analyzed using a TSKgel Protein C4-300 RP-HPLC column (4.6 mm × 150 mm, 3 μm, Tosoh Bioscience LLC., Japan) on a Shimadzu LC-20A System (Shimadzu, Japan) at room temperature. The mobile phase A was pure water with 0.1% TFA, and mobile phase B was pure acetonitrile with 0.1% TFA. Each sample of 20 μl was analyzed at a linear gradient of 40–55% B (v/v) over 5 min, and then 55%–75% B over 20 min at a flow rate of 1 ml/min with UV detection at 280 and 215 nm.

### 2.10 *In Vitro* Bioactivity Assay

The *in vitro* bioactivity of C15-rhG-CSF was determined by NFS-60 cell proliferation using the 3-(4,5-dimethylthiazol-2-yl)-2,5-diphenyl tetrazolium bromide (MTT) assay (Behi et al., 2018). The unmodified rhG-CSF and PEG_10k_-rhG-CSF were used as controls. The NFS-60 cells, which were incubated in RPMI medium 1,640 containing 12.5% horse serum and 2.5% fetal bovine serum in a humidified incubator with 95% air, 5% CO_2_ at 37°C, were washed three times with RPMI medium 1,640 and adjusted to a density of 2.0×10^5^ cells/ml. Then, adjusted cells of 50 μl were loaded into duplicate wells of a 96-well plate, and the samples of rhG-CSF, PEG_10k_-rhG-CSF and C15-rhG-CSF were added. Subsequently, the plate was incubated at 37°C in a 5% CO_2_ atmosphere for 48 h, and then MTT of 20 µl was added and further incubated under the same conditions for 4 h. Then, 100 µl of SDS solution (25%) was added to each well, and the plate was incubated overnight at room temperature to dissolve the formed formazan crystals. The absorbance at 570 nm was measured by a microplate reader (model 550, Bio-Rad, United States).

### 2.11 Pharmacokinetic Properties in Mice

Male KM mice (16–20 g) were randomly allocated into three groups (rhG-CSF, PEG_10k_-rhG-CSF and C15-rhG-CSF groups), and the method of subcutaneous administration and sampling time was shown in Supplementary Table S1. Groups of mice were subcutaneously injected with rhG-CSF, PEG_10k_-rhG-CSF and C15-rhG-CSF at a single dose of 1.0 mg rhG-CSF/kg body weight. After injection, blood samples were collected from orbit at selected times (n = 6 mice at each time) and mice were sacrificed by neck break. The blood samples were immediately centrifuged at 4,000 × *g* for 30 min at 4°C, and then the plasma samples were stored at −20°C until use. The rhG-CSF concentrations of plasma samples were measured using Human G-CSF DuoSet ELISA Kit (R&D Systems, Inc. United States), according to the manuals from the manufacturer. Pharmacokinetic parameters were calculated using DAS 2.0 software (Drug and Statistics Software, Wuhu, China).

### 2.12 *In Vivo* Efficacy in Mice

Male KM mice (16–20 g) except normal control group were intraperitoneally injected with 0.2 ml cyclophosphamide (CTX) at a dose of 1.0 mg rhG-CSF/10 g body weight for three consecutive days, and mice (16–20 g) in normal control group were intraperitoneally injected with equal volume of saline. To determine whether the modeling was successful, five mice from normal control group and model group were randomly selected to measure the white blood cell (WBC) counts. The successfully modeled mice were randomly allocated into seven groups with 12 mice in each group. The method of subcutaneous administration was shown in Supplementary Table S2. The groups were: normal control group, model control group, low-dose rhG-CSF group, high-dose rhG-CSF group, low-dose PEG_10k_-rhG-CSF group, high-dose PEG_10k_-rhG-CSF group, low-dose C15-rhG-CSF group and high-dose C15-rhG-CSF group. The mice in low- and high-dose rhG-CSF group were subcutaneously injected with multiple doses (five times) of 0.1 and 0.2 mg rhG-CSF/kg body weight, respectively, for five consecutive days. The mice in low- and high-dose PEG_10k_-rhG CSF and C15-rhG-CSF group were subcutaneously injected with single dose of 0.5 and 1.0 mg rhG-CSF/kg body weight, respectively. The mice in normal control group and model control group were subcutaneously injected with multiple doses (five times) of saline for five consecutive days. On day 6 after the D1 administration, the mice were sacrificed by neck break, and then the blood samples were collected and the WBC counts were measured using an automatic blood cell analyzer (Mindray BC-2800, China).

### 2.13 Statistical Analysis

Data were expressed as mean ± SD. Statistical inferences were conducted using Student’s t-test by GraphPad Prism six software (GraphPad Software, San Diego, CA, United States). Two-sided values of *p < 0.05, **p < 0.01 and ***p < 0.001 were considered significant for intergroup comparisons.

## 3 Results and Discussion

### 3.1 Synthesis and Characterization of C15-MAL

Since maleimide can highly specific react with the thiol group (Hao et al., 2006), C15-MAL was firstly synthesized for the site-specific fatty chain-modification of rhG-CSF at Cys18 position. The crude C15-MAL was purified using preparative liquid chromatography. The purified C15-MAL achieved a total yield of 41.3% and an HPLC purity of 98.0% (Supplementary Figure S1A), respectively. MALDI-TOF-MS and ^1^H NMR were used to further characterize the C15-MAL. As shown in Supplementary Figure S1B, the molecular weight of the C15-MAL was 307 Da, which was consistent with the theoretical value. As shown in Supplementary Table S3, the ^1^H-NMR spectrum showed that the proton resonance signals (0.91, 1.30, 1.57, 3.49, 6.81) were corresponding to main chemical groups in C15-MAL. Therefore, C15-MAL was successfully prepared.

### 3.2 Modification of rhG-CSF With C15-MAL

**FIGURE 2:**
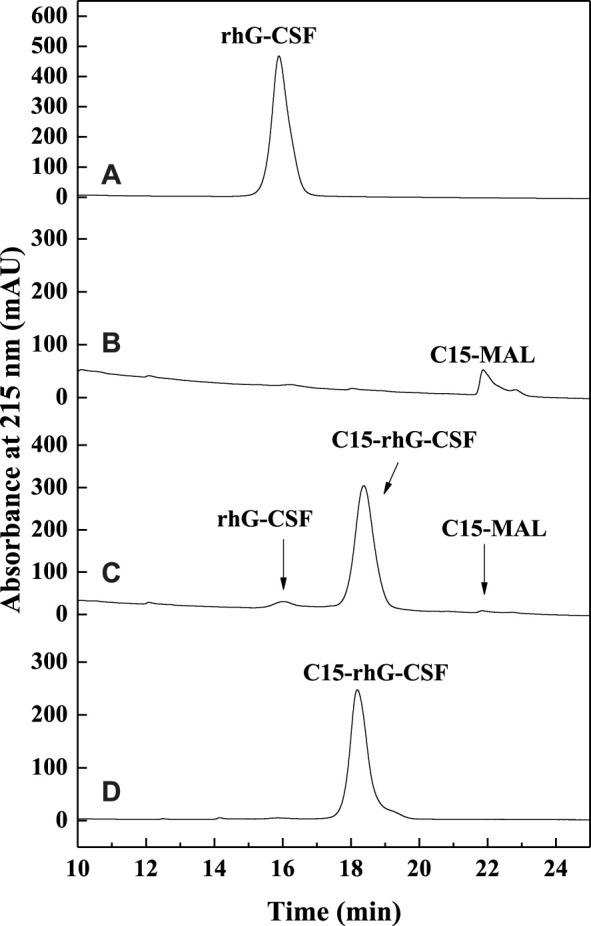
The RP-HPLC analysis of the reaction mixture of the modification of rhG-CSF with C15-MAL. **(A)** rhG-CSF; **(B)** C15-MAL; **(C)** Reaction mixture; **(D)** Purified C15-rhG-CSF. Reaction conditions: rhG-CSF (1 mg/ml) and C15-MAL (mole ratio of C15-MAL to rhG-CSF 5:1) were reacted in 10:1 (v/v) DMSO-phosphate buffer (pH 7.0) at 20°C for 30 min.

**FIGURE 3:**
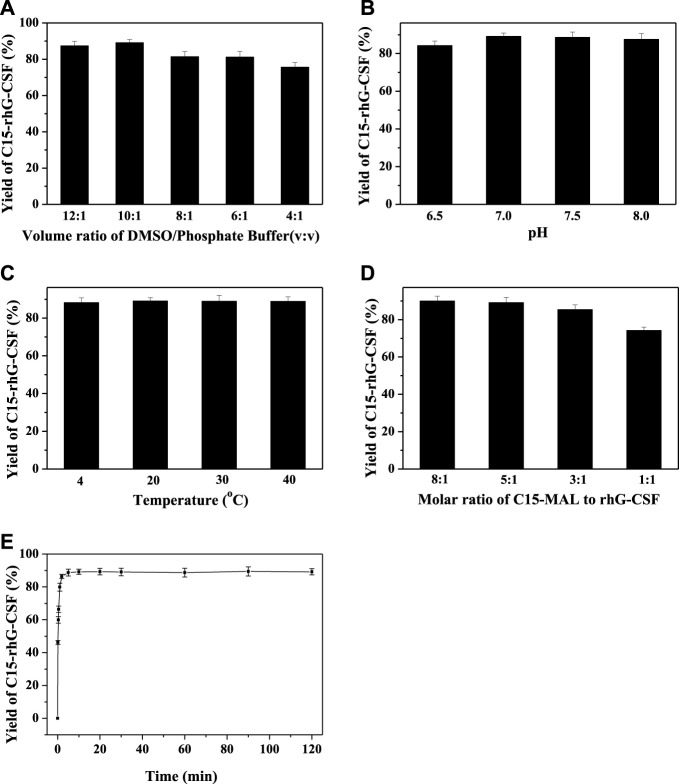
Effects of volume ratio of DMSO/phosphate buffer **(A)**, pH **(B)**, temperature **(C)**, molar ratio of C15-MAL to rhG-CSF **(D)** and reaction time **(E)** on the conjugation of rhG-CSF with C15-MAL.

To achieve an efficient process for the modification of rhG-CSF with C15-MAL, two issues should be addressed: 1) rhG-CSF is highly hydrophilic, while C15-MAL is highly hydrophobic. 2) the free thiol group of Cys 18 in rhG-CSF is buried in a hydrophobic cavity of the protein in aqueous solution. Therefore, suitable solvent systems should be selected to achieve high solubility of the two substrates as well as the adequate exposure of the buried thiol group of Cys 18 of rhG-CSF to react with C15-MAL. One previous study (Peng et al., 2014) reported that PEGylation of rhG-CSF with PEG-MAL in DMSO solvent showed significantly higher reaction rate and PEG-rhG-CSF yield than those obtained in aqueous solvent. Moreover, the secondary structure of rhG-CSF in DMSO solvent could restore its correct form after being PEGylated and separated into the aqueous solution. Another previous study on the fatty chain modification of HV2 with PAL-BTA (Wang et al., 2019) showed that both the two substrates had desirable solubility in mixed aqueous-DMSO solutions. Therefore, a mixed aqueous-DMSO solution was attempted to use as the solvent system for the modification of rhG-CSF with C15-MAL. The mixed DMSO-phosphate buffer solution was used in this study. The reaction mixtures of rhG-CSF with C15-MAL were analyzed by RP-HPLC. As shown in Figure 2C, the different peaks were effectively separated, and the peaks of rhG-CSF and C15-MAL were identified according to their retention times of the corresponding standards (Figures 2A,B). The product peak at a retention time of 18.6 min was collected and then identified as C15- rhG-CSF by MALDI-TOF-MS, and rhG-CSF was used as a control (Supplementary Figure S2). The effects of different reaction conditions on the reaction yield of C15- rhG-CSF were investigated (Figure 3). As shown in Figure 3A, the reaction yield of C15- rhG-CSF firstly increased with the increasing volume ratio of DMSO/phosphate buffer between 4:1 and 10:1, and then a plateau was reached between 10:1 and 12:1. As shown in Figure 3B, as the pH increased, the reaction yield of C15- rhG-CSF increased between pH 6.5 and 7.0, and then slightly decreased between pH 7.0 and 8.0. As shown in Figure 3C, the reaction yield of C15-rhG-CSF presented no difference between 4 and 40°C, indicating that the reaction could be carried out at room temperature without strict control. As shown in Figure 3D, as the molar ratio of C15-MAL to rhG-CSF increased, the reaction yield of C15-rhG-CSF increased between 1:1 and 5:1, and then a plateau was reached between 5:1 and 8:1. As shown in Figure 3E, the reaction was completed within 10 min. This is probably due to the unique feature of DMSO, which can improve the rates of nucleophilic addition reactions, e.g., the PEGylation of rhG-CSF with PEG-MAL in DMSO solvent was completed within 5 min in a previous study (Peng et al., 2014).

In summary, optimized conditions for the modification of rhG-CSF with C15-MAL were the volume ratio of DMSO/phosphate buffer of 10:1, mole ratio of C15-MAL to rhG-CSF of 5:1, pH 7.0, temperature 20°C and reaction time 10 min, consequently, a C15-rhG-CSF yield of 89.2% was achieved.

### 3.3 Purification of C15-rhG-CSF

**FIGURE 4:**
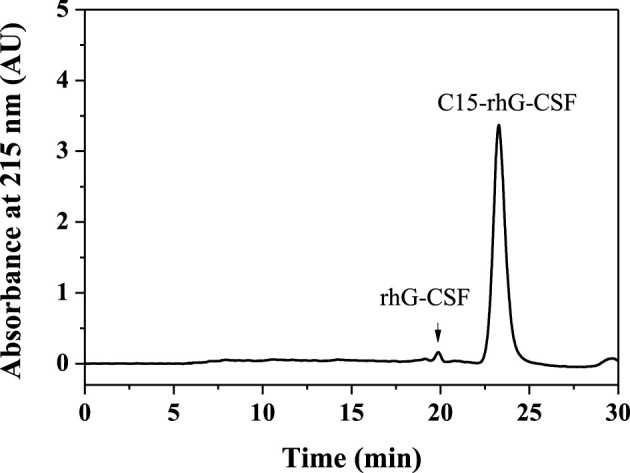
Purification of C15-rhG-CSF by preparative liquid chromatography.

As shown in Figure 4, the reaction mixture obtained from the above optimized conditions was effectively separated and purified using preparative liquid chromatography. The maximum elution peak was collected and analyzed by RP-HPLC, which was verified as the desired product by comparison of its retention time with that of C15-rhG-CSF in Figure 2C. Then, the collected fraction was post-treated by procedures including removal of acetonitrile, dialysis, and concentration, which achieved a very high HPLC purity of 99.0 (Figure 2D) and a yield of 84.6%. PEG_10k_-rhG-CSF was also prepared as control, which was purified using cation exchange chromatography (Supplementary Figure S3), and a HPLC purity of 96.2 was achieved (Supplementary Figure S4).

### 3.4 Characterization of C15-rhG-CSF

#### 3.4 1 CD Spectroscopy Analysis

**FIGURE 5:**
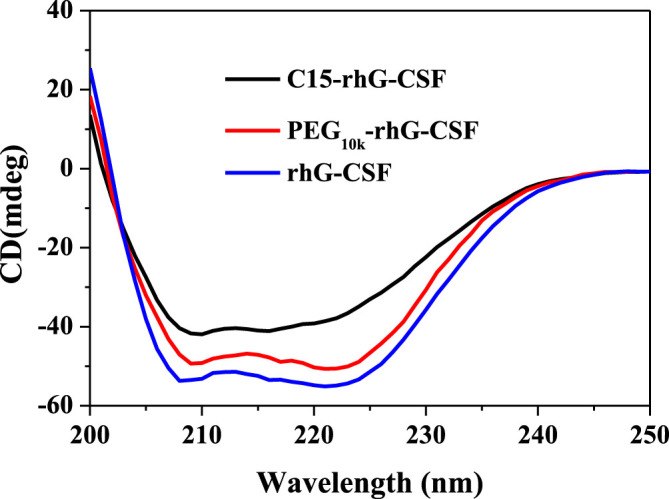
Analysis of rhG-CSF, PEG_10k_-rhG-CSF and C15-rhG-CSF by circular dichroism spectroscopy analysis.

The secondary structures of rhG-CSF, PEG_10k_-rhG-CSF and C15-rhG-CSF were analyzed by CD spectroscopy. As shown in Figure 5, the secondary structures of PEG_10k_-rhG-CSF and C15-rhG-CSF had no significant changes compared with that of rhG-CSF. This indicated that rhG-CSF being coupled with a fatty chain (C15) had no significant effect on its secondary structure. Moreover, C15-rhG-CSF could restore the secondary structure of rhG-CSF after being purified into the aqueous solution. Similar result was observed during the PEGylation of rhG-CSF with PEG-MAL in DMSO solution (Peng et al., 2014).

### 3.5 *In Vitro* Bioactivity Analysis

**FIGURE 6:**
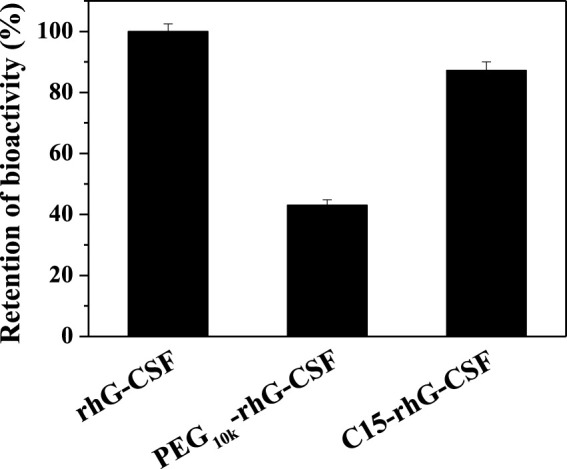
*In vitro* bioactivities of rhG-CSF, PEG-rhG-CSF and C15-rhG-CSF.

The *in vitro* bioactivities of rhG-CSF, PEG_10k_-rhG-CSF and C15-rhG-CSF were measured by NFS-60 cell proliferation using MTT assay. As shown in Figure 6 **,** PEG_10k_-rhG-CSF remained 43.1% of the unmodified G-CSF, which was consistent with the result reported in a previous study (Peng et al., 2014). Relatively, C15-rhG-CSF remained 87.2% of the unmodified G-CSF, which was significantly higher than that of PEG_10k_-rhG-CSF. This was because the coupled PEG_10k_ chain displayed a higher steric resistance on the interaction between rhG-CSF and its receptor than that of the coupled fatty chain.

### 3.6 Pharmacokinetic Properties in Mice

**FIGURE 7:**
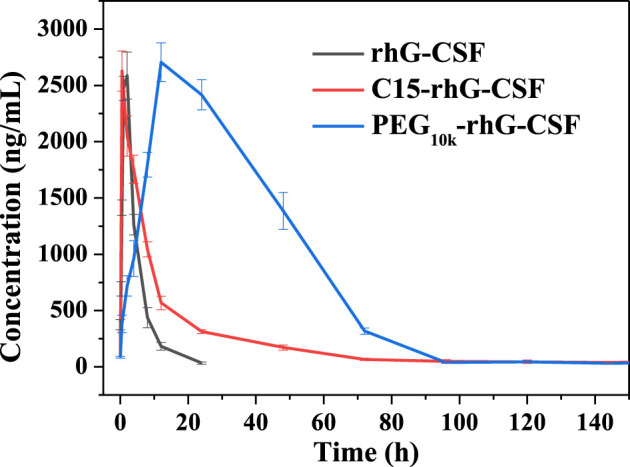
Pharmacokinetic profiles of rhG-CSF, PEG_10k_-rhG-CSF and C15-rhG-CSF.

The pharmacokinetic properties of rhG-CSF, PEG_10k_-rhG-CSF and C15-rhG-CSF were investigated in mice. The rhG-CSF serum concentrations were measured using rhG-CSF -specific ELISA kit following a single subcutaneous injection. The rhG-CSF serum concentration–time curves and the pharmacokinetic parameters were shown in Figure 7 and Table 1. The serum half-life of rhG-CSF was 2.74 ± 0.33 h. In contrast, the serum half-life of C15-rhG-CSF was 5.72 ± 0.43 h, which was 2.1-fold higher than that of rhG-CSF. The AUC_(0-t)_ of C15-rhG-CSF was also 1.8-fold higher than that of rhG-CSF. These results demonstrated that fatty chain modification could prolong serum half-life and increase bioavailability of rhG-CSF. Moreover, serum concentration of C15-rhG-CSF reached peak was 2.15 ± 0.21 h, which was faster than that of rhG-CSF (2.15 ± 0.21 h). In contrast, serum concentration of C15-rhG-CSF reached peak was 11.87 ± 0.32 h, which was slower than that of rhG-CSF. This result indicated that fatty chain modification could promote the trans-membrane absorption/permeability of rhG-CSF after subcutaneous injection. On the other hand, the serum half-life of C15-rhG-CSF was shorter than that of PEG_10k_-rhG-CSF. To develop more long-acting fatty chain-rhG-CSF conjugates, the systematic investigation of the effects of the fatty chain properties (e.g., different types and chain lengths) (Zhu et al., 2018) and the linker between fatty chain and protein (Cho et al., 2020) should be considered in further studies.

**TABLE 1:** Pharmacokinetic parameters of subcutaneous administration of rhG-CSF, PEG_10k_-rhG-CSF and C15-rhG-CSF in mice.

Parameters^a^	rhG-CSF	PEG_10k_-rhG-CSF	C15-rhG-CSF
T_1/2_ (h)	2.74 ± 0.33	13.05 ± 0.45	5.72 ± 0.43
AUC_(0-t)_ (ng·h·mL^−1^)	13,692.70 ± 307.98	124,710.57 ± 7,418.19	24,322.13 ± 1,075.57
C_max_ (ng·mL^−1^)	2,632.42 ± 159.28	2,705.12 ± 157.05	2,626.72 ± 163.01
T_peak_ (h)	2.15 ± 0.21	11.87 ± 0.32	0.52 ± 0.11
CL/f (s) (L·h^−1^·kg^−1^)	1.46 ± 0.03	0.16 ± 0.01	0.85 ± 0.03
V/f (c) (L·kg^−1^)	5.76 ± 0.72	2.97 ± 0.22	6.96 ± 0.50

a Note: T_1/2_: Serum half-life; AUC_(0-t)_: Area under drug concentration vs. time curve; C_max_: Maximal drug concentration; T_peak_: Time of maximal drug concentration; CL/f (s):Clearance over bioavailability; V/f (c):Apparent volume of distribution.

### 3.7 *In Vivo* Efficacy in Mice

**FIGURE 8:**
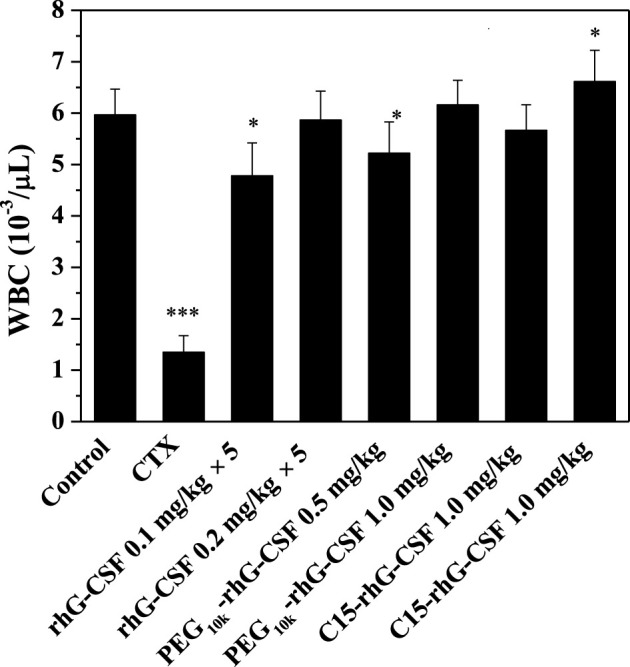
Effects of rhG-CSF, PEG-rhG-CSF and C15-rhG-CSF on the WBC counts in CTX treated mice. The two-sided values of ∗*p* < 0.05, ∗∗*p* < 0.01 and ∗∗∗*p* < 0.001 were considered significant for intergroup comparisons with the normal control group.

The *in vivo* efficacies of rhG-CSF, PEG_10k_-rhG-CSF and C15-rhG-CSF were evaluated based on their effects on the WBC counts in CTX treated mice on day 6 after the D1 administration (Figure 8). The mean WBC count of model control group mice was significantly lower (*p* < 0.001) than that of normal control group mice, indicating that the modeling of mice treated with CTX was successful. The mean WBC counts of the mice in all six experience groups were significantly increased, compared with model control group mice, indicating that all the rhG-CSF, PEG_10k_-rhG-CSF and C15-rhG-CSF had *in vivo* efficacies. The mean WBC counts of the mice in all three experimental high-dose groups were significantly higher than those of all three experimental low-dose groups, respectively, which showed dose-dependent manners. Compared with normal control group mice, the mice in both the single-dose PEG_10k_-rhG-CSF groups and multiple doses rhG-CSF groups at equal total dose of 1.0 mg rhG-CSF/kg body weight showed no significant differences in the mean WBC counts, indicating an equivalent *in vivo* efficacy between PEG_10k_-rhG-CSF by single-dose administration and rhG-CSF by multiple doses administration at the same total dose. Interestingly, the mean WBC counts of the mice in both low- and high-dose C15-rhG-CSF groups were significantly increased, compared with PEG_10k_-rhG-CSF groups, which indicated that C15-rhG-CSF had a better *in vivo* efficacy than that of PEG_10k_-rhG-CSF, although the serum half-life of C15-rhG-CSF was lower than that of PEG_10k_-rhG-CSF (Table 1). This was probably because C15-rhG-CSF had a higher retained *in vitro* bioactivity and better trans-membrane absorption/permeability than those of PEG_10k_-rhG-CSF. Therefore, *in vivo* efficacies of C15-rhG-CSF and PEG_10k_-rhG-CSF were related to their retained *in vitro* bioactivity, serum half-life, trans-membrane absorption/permeability, etc (Menacho-Melgar et al., 2019; Fattahi et al., 2020), and their balances should be considered. Overall, C15-rhG-CSF by single-dose subcutaneous administration had better *in vivo* efficacy than those of both PEG_10k_-rhG-CSF by single-dose administration and rhG-CSF by multiple doses administration at the experimental doses. To deeply evaluate the potential of C15-rhG-CSF to be developed as a candidate drug, its *in vivo* pharmacodynamics and safety (e.g., toxicology and immunogenicity) should be investigated in future studies.

## 4 Conclusion

In this study, we successfully developed a strategy for the site-specific modification of rhG-CSF with C15-MAL in mixed DMSO-aqueous solutions, in which both of the two substrates had high solubility and the thiol group of Cys18 located in a hydrophobic cavity of rhG-CSF could be adequately exposed to react with C15-MAL. Effects of volume ratio of DMSO/phosphate buffer, pH, temperature, molar ratio of C15-MAL to rhG-CSF and reaction time on the conjugation reaction were investigated and optimized. Consequently, the desired product (C15-rhG-CSF) achieved a high yield of 89.2% under the optimized conditions of volume ratio of DMSO/phosphate buffer 10:1, mole ratio of C15-MAL to rhG-CSF 5:1, pH 7.0, temperature 20°C and reaction time 10 min. The C15-rhG-CSF was efficiently separated from the reaction mixture by preparative liquid chromatography, resulting in a high HPLC purity of 99.0% and a yield of 84.6%. Circular dichroism spectroscopy analysis showed that there was no significant change in the secondary structure of C15-rhG-CSF compared with unmodified rhG-CSF. C15-rhG-CSF retained 87.2% of *in vitro* bioactivity of unmodified rhG-CSF. The pharmacokinetic study showed that the half-life of C15-rhG-CSF in mice was 5.72 ± 0.43 h, which was 2.08-fold longer than that of unmodified rhG-CSF. Furthermore, C15-rhG-CSF by single-dose subcutaneous administration showed better *in vivo* efficacy than those of both PEG10k-rhG-CSF by single-dose administration and rhG-CSF by multiple doses administration. This study showed the potential of C15-rhG-CSF to be developed as a candidate drug as well as an efficient process for site-specific fatty chain conjugation of protein and peptide drugs.

## Data Availability

The original contributions presented in the study are included in the article/Supplementary Material, further inquiries can be directed to the corresponding authors.
