# Supplementary material for: Preparation and Characterization of Site-Specific Fatty Chain-Modified Recombinant Human Granulocyte Colony Stimulating Factor
Source: Front Bioeng Biotechnol. 2022 May 23;10:923059. doi: 10.3389/fbioe.2022.923059 (PMC9168434; doi:10.3389/fbioe.2022.923059)
Supplement: Supplementary file 1 [file DataSheet1.docx]

**Supplementary Table S1.** Method of subcutaneous administrationand sampling for the *in vivo* pharmacokinetic study.

| Sampe | Mice (n) | Dose (mg/kg) | Sampling time (h) |
| --- | --- | --- | --- |
| rhG-CSF | 60 | 1.0 | 0, 0.083, 0.25, 0.5, 1, 2, 4, 8, 12, 24 |
| PEG_10k_-rhG-CSF | 78 | 1.0 | 0, 0.5, 2, 4, 8, 12, 24, 48, 72, 96, 120, 144, 168 |
| C15-rhG-CSF | 78 | 1.0 | 0, 0.5, 2, 4, 8, 12, 24, 48, 72, 96, 120, 144, 168 |

**Supplementary Table S2.** Method ofsubcutaneous administrationof rhG-CSF, PEG_10k_-rhG-CSF and C15-rhG-CSF for the *invivo* efficacy study.

| Group | Mice (n) | Dose (mg/kg) | | Administrationtime (day) |
| --- | --- | --- | --- | --- |
| Normal control group | 12 | | - | D1, D2, D3, D4, D5 |
| Model control group | 12 | | - | D1, D2, D3, D4, D5 |
| Low-doserhG-CSF- | 12 | | 0.1 *5 | D1, D2, D3, D4, D5 |
| High-doserhG-CSF | 12 | | 0.2 *5 | D1, D2, D3, D4, D5 |
| Low--dose PEG_10k_ -rhG-CSF | 12 | | 0.5 | D1 |
| High-dose PEG_10k_ -rhG-CSF | 12 | | 1.0 | D1 |
| Low--dose C15 -rhG-CSF | 12 | | 0.5 | D1 |
| High-dose C15 -rhG-CSF | 12 | | 1.0 | D1 |

**Note:** “-”expressed that the mice in normal control group and model control group were subcutaneously injected withequal volume of saline.

“D1, 2,3,…”expressed that the injection was carried out at the first, second, and third,…day.

**Supplementary Table S3.** ^1^H-NMR data of C15-MAL.

| Chemical shift | Chemical group |
| --- | --- |
| 0.91 | -CH_3_ |
| 1.30 | -CH_2_- |
| 1.57 |  |
| 3.49 |  |
| 6.81 |  |

A

B

**Supplementary Figure S1.** Analysis of C15-MAL by RP-HPLC (A) and [MALDI-](https://www.baidu.com/link?url=L8pPmJxcZbI2-dToJ5PuNV-bfiDbLOIJ0YQUCEfZ9YHT70qJ_u_Wzk9KDgD4twK1TXQa8tRq2WI2Np91hpC_aTvvgt6uqPj2OutXOrlAq97&wd=&eqid=b63317d10024e066000000046259f604)TOF-MS (B).

**A**

**B**

**Supplementary Figure S2.** MALDI-TOF-MS analysis of rhG-CSF (A) and C15-rhG-CSF (B).

**Supplementary Figure S3.** Purification of PEG_10k_-rhG-CSF by cation exchange chromatography.

**Supplementary Figure S4.** RP-HPLC analysis of purified PEG_10k_-rhG-CSF.
